# Optimizing transseptal puncture guided by three-dimensional mapping: the role of a unipolar electrogram in a needle tip

**DOI:** 10.1093/europace/euae098

**Published:** 2024-04-15

**Authors:** Yifan Chen, Xiaoyan Wu, Mengting Yang, Zhibin Li, Ruya Zhou, Weiqian Lin, Cheng Zheng, Youdong Hu, Jin Li, Yuechun Li, Jiafeng Lin, Mark M Gallagher, Jia Li

**Affiliations:** Department of Cardiology, Second Affiliated Hospital and Yuying Children’s Hospital of Wenzhou Medical University, No.109 Xueyuan West Road, Lucheng District, Wenzhou 325000, Zhejiang, China; Department of Cardiology, Second Affiliated Hospital and Yuying Children’s Hospital of Wenzhou Medical University, No.109 Xueyuan West Road, Lucheng District, Wenzhou 325000, Zhejiang, China; Department of Cardiology, Second Affiliated Hospital and Yuying Children’s Hospital of Wenzhou Medical University, No.109 Xueyuan West Road, Lucheng District, Wenzhou 325000, Zhejiang, China; Department of Cardiology, Second Affiliated Hospital and Yuying Children’s Hospital of Wenzhou Medical University, No.109 Xueyuan West Road, Lucheng District, Wenzhou 325000, Zhejiang, China; Department of Cardiology, Lishui People’s Hospital, Lishui 323000, China; Department of Cardiology, Second Affiliated Hospital and Yuying Children’s Hospital of Wenzhou Medical University, No.109 Xueyuan West Road, Lucheng District, Wenzhou 325000, Zhejiang, China; Department of Cardiology, Second Affiliated Hospital and Yuying Children’s Hospital of Wenzhou Medical University, No.109 Xueyuan West Road, Lucheng District, Wenzhou 325000, Zhejiang, China; Department of Cardiology, Second Affiliated Hospital and Yuying Children’s Hospital of Wenzhou Medical University, No.109 Xueyuan West Road, Lucheng District, Wenzhou 325000, Zhejiang, China; Department of Cardiology, Second Affiliated Hospital and Yuying Children’s Hospital of Wenzhou Medical University, No.109 Xueyuan West Road, Lucheng District, Wenzhou 325000, Zhejiang, China; Department of Cardiology, Second Affiliated Hospital and Yuying Children’s Hospital of Wenzhou Medical University, No.109 Xueyuan West Road, Lucheng District, Wenzhou 325000, Zhejiang, China; Department of Cardiology, Second Affiliated Hospital and Yuying Children’s Hospital of Wenzhou Medical University, No.109 Xueyuan West Road, Lucheng District, Wenzhou 325000, Zhejiang, China; Cardiology Clinical Academic Group, St George’s University Hospitals NHS Foundation Trust, Blackshaw Road, London SW17 0QT, UK; Department of Cardiology, Second Affiliated Hospital and Yuying Children’s Hospital of Wenzhou Medical University, No.109 Xueyuan West Road, Lucheng District, Wenzhou 325000, Zhejiang, China

**Keywords:** Transseptal puncture, Three-dimensional electroanatomic mapping, Unipolar electrogram, Current of injury, Foramen ovalis, Zero fluoroscopy

## Abstract

**Aims:**

A three-dimensional electroanatomic mapping system–guided transseptal puncture (3D-TSP), without fluoroscopy or echocardiography, has been only minimally reported. Indications for 3D-TSP remain unclear. Against this background, this study aims to establish a precise technique and create a workflow for validating and selecting eligible patients for fluoroless 3D-TSP.

**Methods and results:**

We developed a new methodology for 3D-TSP based on a unipolar electrogram derived from a transseptal needle tip (UEGM tip) in 102 patients (the derivation cohort) with intracardiac echocardiography (ICE) from March 2018 to February 2019. The apparent current of injury (COI) was recorded at the muscular limbus of the foramen ovalis (FO) on the UEGM tip (sinus rhythm: 2.57 ± 0.95 mV, atrial fibrillation: 1.92 ± 0.77 mV), which then disappeared or significantly reduced at the central FO. Changes in the COI, serving as a major criterion to establish a 3D-TSP workflow, proved to be the most valuable indicator for identifying the FO in 99% (101/102) of patients compared with three previous techniques (three minor criteria) of reduction in atrial unipolar or bipolar potential and FO protrusion. A total of 99.9% (1042/1043) patients in the validation cohort underwent successful 3D-TSP through the workflow from March 2019 to July 2023. Intracardiac echocardiography guidance was required for 6.6% (69/1042) of patients. All four criteria were met in 740 patients, resulting in a 100% pure fluoroless 3D-TSP success rate.

**Conclusion:**

In most patients, fluoroless 3D-TSP was successfully achieved using changes in the COI on the UEGM tip. Patients who met all four criteria were considered suitable for 3D-TSP, while those who met none required ICE guidance.

## Graphical Abstract

**Graphical Abstract:**
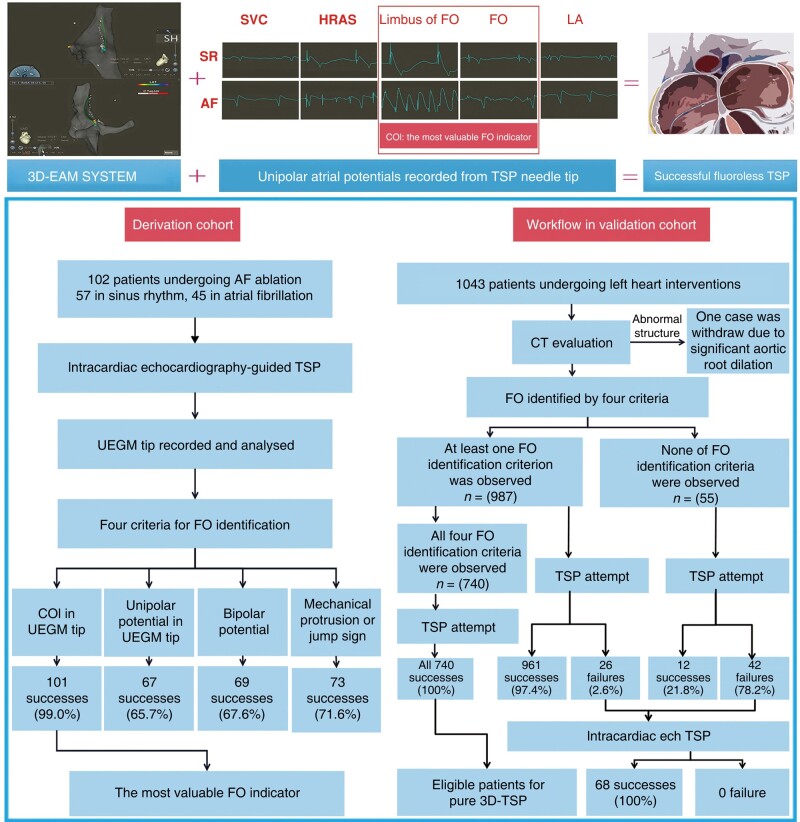
Flow diagram and summary of the study. Our study revealed distinct morphologies of a unipolar electrogram recorded from a transseptal needle tip at different atrial locations, particularly emphasizing the occurrence of sudden disappearance or marked diminution in the amplitude of the COI during needle tip withdrawal from the muscular limbus to the FO. The FO indicators, especially the changes in the COI, permitted precise FO localization, enabling safe and successful 3D-EAM system-guided TSP without fluoroscopy. AF, atrial fibrillation; COI, current of injury; CT, computed tomography; 3D-EAM system, three-dimensional electroanatomic mapping system; FO, foramen ovalis; HRAS, high right atrial septum; Limbus, the muscular limbus of the foramen ovalis; SR, sinus rhythm; SVC, superior vena cava; TSP, transseptal puncture; UEGM tip, a unipolar electrogram derived from a transseptal needle tip.

**What’s new?**

- This study revealed distinct patterns of a unipolar electrogram derived from a transseptal needle tip (UEGM tip) obtained at different atrial sites, as verified by intracardiac echocardiography.
- The changes in the current of injury (COI) served as the most remarkable indicator for identifying the precise position of the central foramen ovalis (FO) among three other criteria, namely unipolar spiked potential, bipolar potential, and FO protrusion.
- The workflow that was mainly based on the changes in the COI led to a safe and successful transseptal puncture guided by a three-dimensional electroanatomic mapping system without fluoroscopy.

## Introduction

Catheter ablation is now considered a first-line treatment for atrial fibrillation (AF), causing a high procedure volume that raises concerns about radiation exposure and orthopaedic injuries among operators.^1–3^ Transseptal puncture (TSP) remains the ultimate milestone towards achieving the complete elimination of fluoroscopy during ablation procedures, despite significant advancements in techniques over recent decades causing considerable reductions in X-ray usage.^4^

Intracardiac echocardiography (ICE) or transoesophageal echocardiography facilitates fluoroless TSP by enabling precise needle localization and direct foramen ovalis (FO) visualization.^5–7^ However, cost constraints, additional venous access requirements, and patient intolerance without general anaesthesia limit their widespread adoption. Thus, a thorough assessment of their necessity remains essential.

Based on the anatomical characteristics of the prominent limbus and thin FO, several studies have revealed the feasibility of performing a three-dimensional electroanatomic mapping system (3D-EAMS)-guided TSP (3D-TSP) without fluoroscopy or ultrasound guidance.^7–12^ However, these studies have not addressed the issue of balance between safety and cost considerations when implementing purely fluoroless 3D-TSP. Therefore, identifying the indications for this approach becomes crucial. Additionally, safety concerns persist because the FO is indirectly localized by surrounding landmarks, bipolar voltage mapping, and mechanical protrusion that is obtained before TSP with a different catheter rather than the moment of puncture. This may introduce potential imprecision caused by map shiftings from patient movement and discrepancies between catheter-based FO localization and needle placement. Thus, precise FO identification in real time is crucial for guiding 3D-TSP.

To address these issues, this study aimed (i) to develop a new method for 3D-TSP based on a real-time acquisition of a unipolar electrogram (UEGM) derived from a transseptal needle tip (UEGM tip) and (ii) to evaluate the feasibility and safety of a workflow with four criteria mainly based on the UEGM tip for purely fluoroless 3D-TSP and identify eligible candidates for 3D-TSP with or without ICE guidance.

## Methods

### Patient population and study design

The study was conducted in two phases: (i) a derivation phase aimed at developing a new method by recording and analysing the UEGM tip at various atrial sites during 3D-TSP with ICE guidance in 102 patients with AF and (ii) a validation phase performed to evaluate the feasibility and safety of a 3D-TSP workflow mainly based on the UEGM tip in 1043 consecutive patients requiring left atrial (LA) interventions and then determine eligible candidates for 3D-TSP (see *Graphical Abstract*). This study excluded patients with an LA thrombus, a cardiac implantable electronic device, or an atrial septal defect occluder. All procedures were completed under conscious sedation, using a single transseptal approach for AF ablation. A UEGM was recorded at a sweep speed of 100 mm/s and typically filtered at 0.5–500 Hz, while a bipolar electrogram was filtered at 30–500 Hz. The study was approved by the Ethics Committee of the Second Affiliated Hospital of Wenzhou Medical University, and all patients provided informed consent.

### A three-dimensional model of the right atrium

An ablation catheter (ThermoCool SmartTouch™; Biosense Webster Inc.) was inserted via the right femoral venous access with an SL1 sheath (Abbott Laboratories). Fast anatomical mapping was used to create a 3D model (CARTO3; Biosense Webster Inc.) of the right atrium with greater detail for the septum, coronary sinus, superior vena cava (SVC), inferior vena cava, and sites with His potential.

### Recording of the unipolar electrogram on a needle tip during transseptal puncture in the derivation cohort

The 3D-TSP procedures were previously described in detail.^7–12^ In brief, a TSP rehearsal involved retracting the ablation catheter from the SVC with the support of an SL1 sheath to locate the FO, monitored by 3D-EAMS and ICE using left anterior oblique projections of 135° and 45°, while simultaneously recording bipolar potentials. Subsequently, the catheter was repositioned within the SVC and replaced with a transseptal needle (BRK-1®; Abbott Laboratories). The proximal end of the needle was connected to two clips connected to a pin box for visualization of the needle tip position^8^ and real-time display of the UEGM tip in 3D-EAMS.^9^

**Figure 1:**
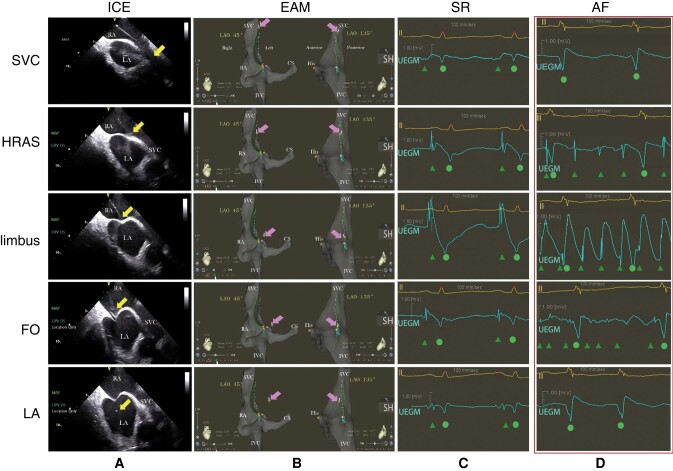
The changes on the UEGM tip during TSP with ICE verification. The entire assembly was pulled down from the SVC to the FO. The needle tip location was confirmed by intracardiac echo (*A*, indicated by the yellow arrows) and visualized by 3D-EAM using left anterior oblique 135° and 45° projections (*B*, indicated by the pink arrows) at various atrial sites, including the SVC, the high right atrial septum, the muscular limbus, the FO, and the LA chamber. Simultaneously, the morphology and amplitude of the unipolar electrogram derived from the needle tip were recorded (*C* for SR and *D* for another case with AF). AF, atrial fibrillation; CS, coronary sinus; EAM, electroanatomic mapping; FO, foramen ovalis; His, His bundle; HRAS, high right atrial septum; ICE, intracardiac echocardiography; IVC, inferior vena cava; Limbus, the muscular limbus of the foramen ovalis; LA, left atrium; RA, right atrium; SR, sinus rhythm; SVC, superior vena cava; (UEGM) tip, (a unipolar electrogram) derived from a transseptal needle tip. Green triangle indicates unipolar atrial potentials recorded from the needle tip. Green circle denotes far-field unipolar ventricular potentials.

With ICE guidance, the needle assembly was withdrawn from the SVC and it reached the FO, where the interatrial septum was tented. After crossing through the FO carefully, the SL1 sheath was advanced 2 cm into the LA over the dilator and needle.^13^ The UEGM morphology and amplitude, initially detected from the distal electrode of the catheter during rehearsal, were subsequently recorded from the needle tip during real TSP while confirming their location at different atrial sites by ICE (*Figure 1*).

### Criteria for identifying the foramen ovalis in a three-dimensional electroanatomic mapping system

The following four criteria, based on previous studies and derivation outcomes, were used to determine the FO when withdrawing the catheter or needle tip from the muscular limbus into the central FO. (i) The major criterion is current of injury (COI) changes on the UEGM tip: sudden disappearance or a significant decrease in amplitude reaches at least a three-fold reduction when withdrawing the needle assembly. The other three minor criteria are (ii) atrial unipolar potential reduction on the UEGM tip: a minimum three-fold amplitude reduction of atrial unipolar potential documented on the UEGM tip when withdrawing the needle assembly; (iii) reduction of atrial bipolar potential: decrease in amplitude reaches at least three-fold during the rehearsal of withdrawing the ablation catheter;^8^ and (iv) mechanical protrusion or jump sign: FO protrusion or jump sign is visible in the EAMS when withdrawing the needle tip or ablation catheter.^10,11^

### A workflow for evaluation and a unipolar electrogram–guided transseptal puncture in the validation cohort

A two-step workflow was applied in the validation cohort to identify eligible patients and assess the feasibility and safety of fluoroless 3D-TSP.

First, a routine conventional (or an ultra-low dose for patients aged ≤18 years) computed tomography (CT) scan was pre-operatively reviewed to evaluate anatomical information, including atrial dimensions, septum orientation, cardiac rotation, and adjacent structures such as aortic root size. The procedure should be assisted by ICE or even cancelled when space is insufficient in the septum to ensure safe 3D-TSP (see Supplementary material online, *Figure S1*).

Secondly, a preview was conducted using an ablation catheter rehearsal followed by the needle assembly to reassess its feasibility before the actual TSP. Patients were categorized based on the preview results into the OFC group, which included patients who met at least one of the four criteria, and the NFC group, which comprised patients who met none of the criteria, with their FO inferred from the surrounding landmarks only. Both groups initially underwent 3D-TSP without additional imaging modalities. Intracardiac echocardiography or fluoroscopy would be sequentially used if TSP failed with three attempts.

Successful 3D-TSP was confirmed by visualizing the needle tip position in the 3D-EAMS and drawing oxygenated blood, further verified by observing UEGM-tip changes, transitioning from near field (with the needle tip tightly engaged with the FO) to far field (with the needle tip inside the LA chamber) during septal crossing. Finally, the SL1 sheath was advanced up to 2 cm into the LA.

All patients were evaluated by echocardiography to exclude pericardial effusion throughout the perioperative period and before discharge.

### Endpoints and adverse events in the validation cohort

The primary endpoints of the validation study were (i) the success rate of 3D-TSP, (ii) the incidence of TSP with three attempts, and (iii) the usage rate of ICE or X-ray. The secondary endpoints included (i) pre-transseptal duration, including right atrial geometry construction and TSP rehearsal, and (ii) transseptal duration, starting from needle tip exposure in the SVC to the LA chamber.

Complications related to TSP comprised pericardial effusion or tamponade, atrial or aortic perforation, cerebral ischaemia, intrathoracic bleeding, and death.

### Statistical analysis

Continuous variables were presented as mean ± standard deviation, while categorical variables were expressed as counts and percentages. A *t*-test was used for the comparison of normally distributed continuous variables; otherwise, the Mann–Whitney *U* test was used. The *χ*^2^ test was used to compare categorical variables. A two-tailed *P*-value of <0.05 indicated statistical significance. Statistical Package for the Social Sciences version 23.0 (IBM Corp., Armonk, NY, USA) was used for all statistical analyses.

## Results

### Patient characteristics

**Figure 2:**
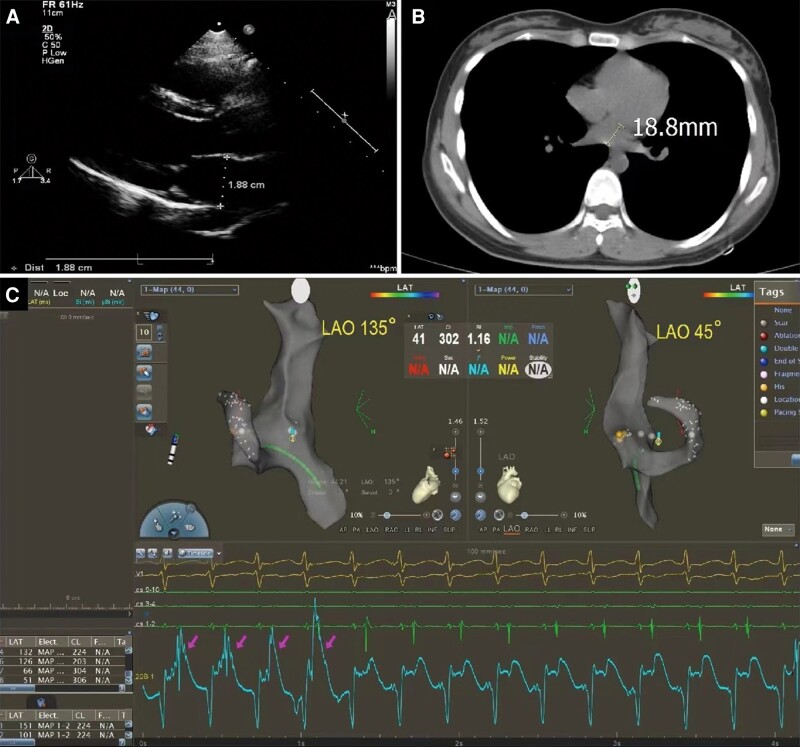
An 8-year-old patient with a small LA underwent fluoroless TSP. The minimum anteroposterior diameter of the left atrium was measured as 18.8 mm using both echocardiography (*A*) and ultra-low-dose CT (*B*). Successful TSPs were guided by EAM and a needle tip unipolar electrogram without the need for fluoroscopic or echocardiographic guidance. High-amplitude atrial potentials with the current of injury (*C*, first four beats, indicated by the pink arrows) could indicate the needle tip location at the muscular limbus of the FO, although in the episode of supraventricular tachycardia. Low-amplitude spiked potentials without the current of injury (*C*, the remaining beats) indicate that the needle tip had reached the central FO. EAM, electroanatomic mapping; FO, foramen ovalis; LA, left atrium; TSP, transseptal puncture.

The derivation cohort consisted of 102 patients with AF (age: 65.6 ± 10.3 years, range: 33–88; 65 men), of whom 57 presented with sinus rhythm (SR), while 45 had AF during TSP. The LA anteroposterior diameter was 40.8 ± 13.6 mm. The validation cohort included 1043 consecutive patients (age: 53.2 ± 14.7 years, range: 8–92; 637 men). Of them, 287, 141, 339, 237, and 39 had left accessory pathway, LA tachycardia, paroxysmal AF, persistent AF, and left ventricular arrhythmia, respectively. The LA diameter was 37.9 ± 19.8 mm, with a minimum value of 18.8 mm (*Table 1* and *Figure 2*).

**Table 1:** Baseline characteristics of patients in the derivation and validation cohorts

Characteristic	Derivation cohort (*n* = 102)	Validation cohort (*n* = 1043)
Age, mean ± SD, year	65.6 ± 10.3	53.2 ± 14.7
Sex		
Male	65 (63.7%)	637 (61.1%)
Arrhythmia		
Paroxysmal AF	57 (55.9%)	339 (32.5%)
Persistent AF	45 (44.1%)	237 (22.7%)
Left pathway		287 (27.5%)
Left AT		141 (13.7%)
Left VA		39 (3.7%)
Prior TSP history	9 (8.8%)	58 (5.6%)
LVEF, mean ± SD, %	61.9 ± 8.9	62.4 ± 6.4
LAD, mean ± SD, mm	40.8 ± 13.6	37.9 ± 19.8

AF, atrial fibrillation; AT, atrial tachycardia; LAD, left atrium diameter; LVEF, left ventricular ejection fraction; SD, standard deviation; TSP, transseptal puncture; VA, ventricular arrhythmia.

### The characteristics of the unipolar electrogram in various atrial regions

Dynamic changes in the morphology and amplitude of the UEGM tip were demonstrated at various atrial sites during TSP (*Figure 1* and *Table 2*). Two key results proved particularly valuable for the safe and successful implementation of TSP in all 102 patients.

**Table 2:** The characteristics of a UEGM tip at various atrial sites in the derivation cohort

Sites	SVC	HRAS	Limbus (potential)	FO (potential)	Limbus (COI)	FO (COI)	LA
Amplitude: SR, mean ± SD, mV	0.21 ± 0.08	1.02 ± 0.38	2.52 ± 0.86^a^	0.37 ± 0.26	2.57 ± 0.95^b^	0.24 ± 0.22	0.23 ± 0.10
Amplitude: AF, mean ± SD, mV	0.20 ± 0.08	0.85 ± 0.33	1.49 ± 0.31^c^	0.26 ± 0.14	1.92 ± 0.77^d^	0.21 ± 0.12	0.23 ± 0.12
Morphology	Blunt	Steep	Steep	Steep	AP-like	Flat	Blunt

AF, atrial fibrillation; AP-like, action potential-like; COI, current of injury; FO, foramen ovalis; HRAS, high right atrial septum; LA, left atrium; Limbus, the muscular limbus of the foramen ovalis; SD, standard deviation; SR, sinus rhythm; SVC, superior vena cava; UEGM tip, a unipolar electrogram derived from a transseptal needle tip.

^a^Atrial unipolar potentials at Limbus vs. FO in SR, *P* < 0.001.

^b^COI at Limbus vs. FO in SR, *P* < 0.001.

^c^Atrial unipolar potentials at Limbus vs. FO in AF, *P* < 0.001.

^d^COI at Limbus vs. FO in AF, *P* < 0.001.

First, a distinct morphology of the COI with high amplitude (2.57 ± 0.95 mV in SR, 1.92 ± 0.77 mV in AF) was recorded when the needle tip was ICE-confirmed at the muscular limbus above the FO. A sudden disappearance or significant reduction of the COI was observed in 99% of patients (101/102) after the needle tip fell into the central FO. A simultaneous shift in atrial unipolar potentials from a high amplitude (2.52 ± 0.86 mV in SR, 1.49 ± 0.31 mV in AF) to a low amplitude (0.37 ± 0.26 mV in SR, 0.26 ± 0.14 mV in AF) was observed, which occurred in 65.7% of patients (67/102, *P* < 0.001).

**Figure 3:**
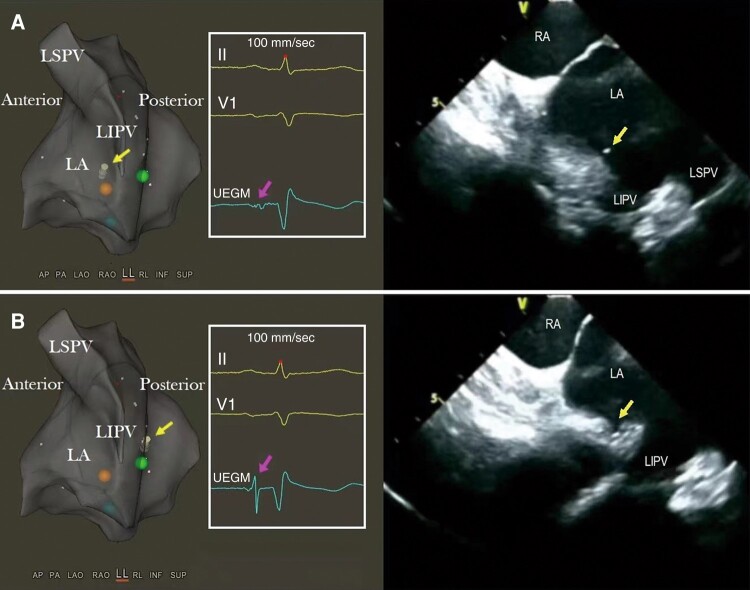
A unipolar electrogram derived from a transseptal needle tip further indicates the needle tip position within the LA. A low-amplitude far-field atrial potential was observed after a successful TSP on the unipolar electrogram (*A*, indicated by the pink arrow) when the needle tip (*A*, indicated by the yellow arrow) resided within the left atrial chamber as confirmed by both the electroanatomic map and the intracardiac echo. Once the needle tip made contact with the left atrium, in this case being the posterior wall (*B*, indicated by the yellow arrow), a high-amplitude near-field spiked atrial potential (*B*, indicated by the pink arrow) reappeared on the unipolar electrogram. At this point, further needle advancement is contraindicated and immediate withdrawal should be performed instead. LA, left atrium; LIPV, left inferior pulmonary vein; LSPV, left superior pulmonary vein; RA, right atrium; TSP, transseptal puncture; UEGM, unipolar electrogram.

Secondly, a transition in the UEGM tip from a spiked near-field to a blunt far-field atrial unipolar potential was observed in all 102 patients when the needle tip penetrated from the FO into the LA chamber. Notably, immediate withdrawal should be performed if a near-field atrial potential with increased amplitude recurs, indicating excessive advancement of the needle tip touching the atrial wall (*Figure 3*).

### Identification of the foramen ovalis by using a unipolar electrogram derived from a transseptal needle tip

**Figure 4:**
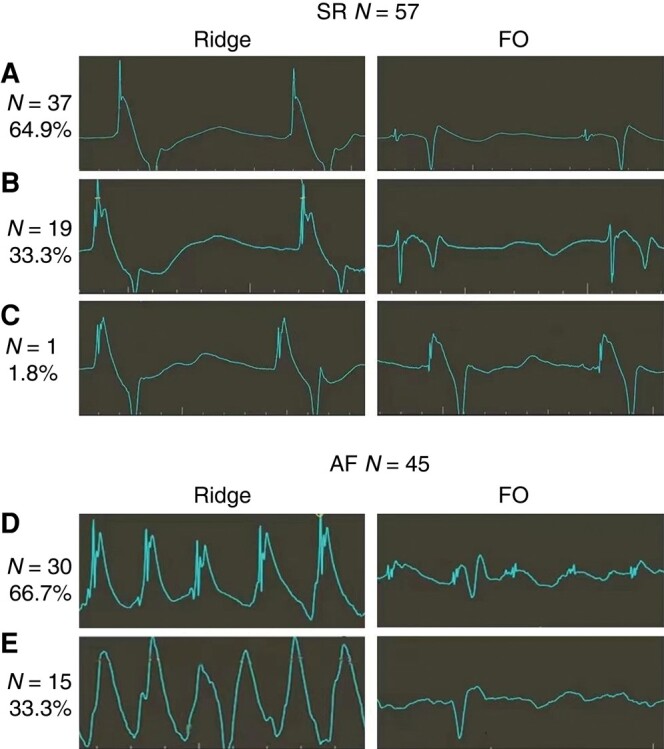
Different subtypes of a UEGM tip from the muscular limbus to the FO. Among the 57 patients in sinus rhythm, 37 patients (64.9%, Group A) experienced distinctive changes in the spiked potentials in the unipolar electrogram as well as the COI. Identification of the FO through amplitude reduction in spiked potentials was unsuccessful in 19 patients (33.3%, Group B), while it remained achievable through COI changes. The FO could not be identified either by changes in the spiked potentials or COI only in one patient (1.8%, Group C). Of 45 patients with AF, 30 (66.7%, Group D) exhibited changes in both the COI and the spiked potentials and 15 (33.3%, Group E) demonstrated the FO that could be identified only through the reduction in the COI but not by changes in the spiked potentials. AF, atrial fibrillation; COI, current of injury; FO, foramen ovalis; UEGM, a unipolar electrogram derived from a transseptal needle tip.

We successfully identified the FO in 98.2% of patients (56/57) with SR based on the main criterion of the changes in the COI (*Figure 4*, Groups A and B). Of these patients, 64.9% (37/57, *Figure 4*, Group A) had FO identified when relying solely on one minor criterion of three-fold amplitude reduction in the spiked atrial unipolar potential on the UEGM tip. Furthermore, FO identification was possible only through the changes in the COI for 19 patients without a reduction in the amplitude of the spiked atrial unipolar potential (*Figure 4*, Group B). Notably, there was one case of failure due to minor changes in either the COI or the spiked atrial potentials (*Figure 4*, Group C, and Supplementary material online, *Figure S2*).

The FO in 45 patients with AF was identified by following the criterion of the changes in the COI. Among them, 66.7% (30/45) demonstrated changes that meet both the COI and the reduced atrial unipolar potential criteria (*Figure 4*, Group D), while the FO could merely be identified through the changes in the COI without any concurrent presence of atrial potentials for the remaining 33.3% of patients (15/45; *Figure 4*, Group E).

Altogether, the changes in the COI on the UEGM tip greatly facilitated FO identification, yielding a success rate of 99% (101/102) compared with 65.7% (67/102, *P* < 0.001), 69.6% (71/102, *P* < 0.001), and 75.5% (77/102, *P* < 0.001) achieved through the analysis of the spiked atrial unipolar potential, atrial bipolar potential, and mechanical protrusion or jump sign in the 3D-EAMS, respectively. The sudden changes in the COI provided a more reliable approach for determining the FO than other methodologies (*Table 3*).

**Table 3:** A comparison of different criteria for FO identification

Criteria for FO identification	Total (*n* = 102)	
	Succeed (%)	Failed (%)
COI on UEGM tip^a^	101 (99.0%)*^,#,&^	1 (1.0%)
Atrial uipolar potential^b^	67 (65.7%)	35 (34.3%)
Mechanical protrusion or jump sign^c^	77 (75.5%)	25 (24.5%)
Atrial bipolar potential^d^	71 (69.6%)	31 (30.4%)

COI, current of injury; FO, foramen ovalis; UEGM tip, a unipolar electrogram derived from a transseptal needle tip.

^a^The criterion for identifying the FO through the current of injury is applied when the disappearance or decrease in amplitude reaches at least a three-fold reduction.

^b^The criterion for identifying the FO through spiked potentials is that its amplitude should be less than one-third of that of the right atrium.

^c^The criterion for identifying the FO through mechanical protrusion is that FO protrusion needle tip jump sign should be visible in the EAMS.

^d^The criterion for identifying the FO through bipolar voltage mapping is that its amplitude should be less than one-third of that of the right atrium.

^*^Compared with the success rate in the spiked potential in the UEGM tip, *P* < 0.001.

^#^Compared with the success rate in mechanical protrusion, *P* < 0.001.

^&^Compared with the success rate in the bipolar potential, *P* < 0.001.

### The outcome of fluoroless transseptal puncture in the validation cohort

**Figure 5:**
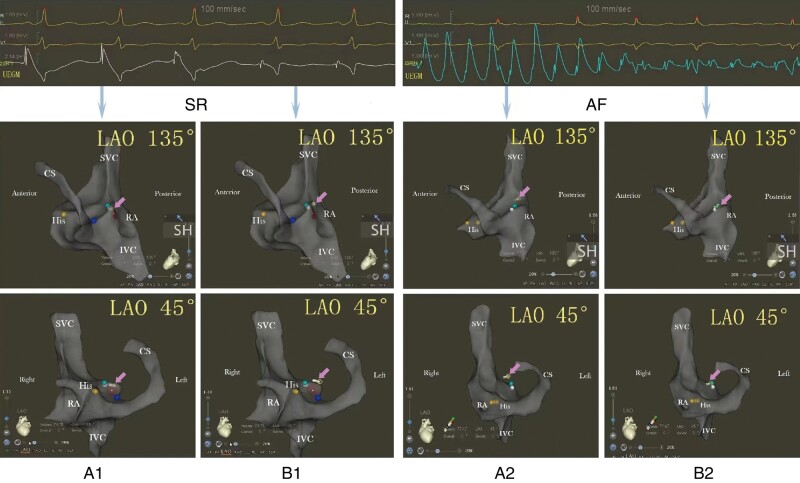
Representative cases demonstrating successful fluoroless TSP utilizing a UEGM tip. Sudden changes in the current of injury could be observed on the unipolar electrogram from the needle tip during its withdrawal from the muscular limbus to the foramen ovalis, regardless of SR or AF origin. High-amplitude atrial potentials with the current of injury (the first three beats in SR and the first eight beats in AF) indicate that the needle tip is located at the muscular limbus (indicated by the pink arrows, *A1* for SR, *A2* for AF), as confirmed on the electroanatomic map in left anterior oblique 135° and 45° projection. Low-amplitude spiked potentials without or low-amplitude current of injury (the last two beats in SR and the last nine beats in AF) indicate that the needle tip has reached the central FO (indicated by the pink arrows, *B1* for SR, *B2* for AF). AF, atrial fibrillation; FO, foramen ovalis; SR, sinus rhythm; TSP, transseptal puncture; UEGM, a unipolar electrogram derived from a transseptal needle tip.

Successful 3D-TSP was performed on the remaining 1042 patients in the validation cohort, except for one cancellation due to a significant dilation of the ascending aorta (see Supplementary material online, *Figure S1* and *Table 4*). Of them, 93.4% (973/1042) required no additional imaging modalities, and only 6.6% (69/1042) needed ICE guidance, with none requiring an X-ray. The initial success rate reached 90.7% (945/1042), with two attempts for 28 and three attempts for 69 patients, of whom 9 had the actual TSP site spatially displaced from the previously tagged FO location by a maximum distance of 12 mm, following which ICE was applied. The pre-transseptal duration and transseptal duration averaged 7.4 ± 3.4 and 1.5 ± 2.1 min, respectively. Transseptal puncture–related pericardial effusion developed in 1 patient (1/1042, 0.096%) during the early stage of the validation phase, which did not require drainage, and no other complications or deaths were observed within 30 days. *Figure 5* and Supplementary material online, *Videos S1* and *S2*, present typical cases demonstrating successful UEGM-tip-guided 3D-TSP in the validation cohort.

**Table 4:** Outcome of UEGM-tip-guided 3D-TSP in the validation cohort

Operation characteristic	Total (*n* = 1043)	OFC group (*n* = 987)	NFC group (*n* = 55)	*P*-value
Successful cases	1042 (99.9%)	987 (100%)	55 (100%)	
Pre-transeptal duration, mean ± SD, min^a^	7.4 ± 3.4	7.3 ± 3.3	8.4 ± 5.8	0.169
Transeptal duration, mean ± SD, min	1.5 ± 2.1	1.5 ± 2.0	3.9 ± 2.3	<0.001
Number of attempts				
1	945 (90.7%)	935 (94.7%)	10 (18.2%)	<0.001
2	28 (2.7%)	26 (2.6%)	2 (3.6%)	0.985
≥3	69 (6.6%)	26 (2.6%)	43 (78.2%)	<0.001
Imagine modalities to guide TSP				
Purely 3D-EAM system	973 (93.4%)	961 (97.4%)	12 (21.8%)	<0.001
ICE assistance	69 (6.6%)	26 (2.6%)	43 (78.2%)	<0.001
Fluoroscopy assistance	0	0	0	
Challenging TSP requiring radiofrequency				
Energy assistance	22 (2.1%)	16 (1.7%)	6 (10.9%)	<0.001
Complications				
Pericardial effusion	1 (0.1%)	0 (0%)	1 (1.8%)	0.053

3D-EAM system, three-dimensional electroanatomic mapping system; 3D-TSP, three-dimensional mapping-guided transseptal puncture; ICE, intracardiac echocardiography; UEGM tip, a unipolar electrogram derived from a transseptal needle tip.

^a^Pre-transeptal duration includes the time of right atrium geometry reconstruction and TSP rehearsal by using the ablation catheter.

### Comparison of transseptal puncture outcome: OFC group vs. NFC group

The FO was identified in 943 (90.5%), 745 (71.5%), 757 (72.4%), and 809 (77.6%) patients through the criterion of the changes in the COI, atrial unipolar or bipolar potential reduction, and jump sign in the 3D-EAMS, respectively. Interestingly, 740 (71.0%) patients met all four criteria, resulting in a 100% success rate for pure 3D-TSP. However, we propose at least one of four criteria to be met as an inclusion criterion for the OFC group, with 94.7% (987/1042) patients enrolled, considering that the other three minor criteria can supplement the main criterion for identifying the FO. Conversely, 5.3% (55/1042) were included in the NFC group without their meeting any criteria. The success rate of fluoroless 3D-TSP without additional imaging modalities was 97.4% (961/987) in the OFC group, including 5 patients with severe cardiac rotational (see Supplementary material online, *Figure S1*) and 1 patient with kyphoscoliosis (see Supplementary material online, *Figure S3*), compared with only 21.8% (12/55, *P* < 0.001) in the NFC group. The OFC group demonstrated a significantly lower TSP incidence with three attempts and ICE usage rate, along with shorter durations for transseptal procedures compared with the NFC group (*Table 4*). Intracardiac echocardiography guidance was required for 69 patients, with 2.6% (26/987) in the OFC group and a significantly higher 78.2% (43/55, *P* < 0.001) in the NFC group.

## Discussion

### Main findings

Our study reveals that a sudden disappearance or significant reduction of the COI on the UEGM tip provides more reliable FO identification, ensuring successful 3D-TSP without fluoroscopy. A workflow for fluoroless 3D-TSP mainly using the UEGM tip has been proved feasible and safe in a large cohort, with 6.6% of patients requiring ICE assistance. Patients who meet all criteria for FO identification are eligible candidates for pure 3D-TSP. Those meeting at least one criterion for FO identification could also successfully undergo pure 3D-TSP and are suitable candidates on whom it could be attempted, considering the factors of safety and cost. Patients meeting none of the four criteria should consider ICE guidance.

### Disappearance of the current of injury exhibited as a more reliable marker to identify the foramen ovalis compared with previous studies

Accurate FO localization during TSP is essential to ensure success and patient safety.^14,15^ Recent studies revealed pure 3D-TSP without auxiliary imaging modalities based on indirect FO localization by surrounding landmarks, bipolar voltage mapping, and mechanical protrusion.^7,8,10,11^ However, some limitations should be considered.

The main limitation of the previously reported 3D-TSP techniques is spatial displacement. Surrounding landmarks may shift because of bodily movements and irregular respiratory or different cardiac cycles, as shown in a recent study in which it was found that premature beats caused a misidentification of the site of origin in the 3D-EAMS.^16^ Similarly, the previously catheter-tagged FO position may not always represent its actual location during subsequent needle puncture. The present study found only nine patients with spatial displacement, but the consequences would be serious if TSP is continued. Therefore, COI disappearance on the UEGM tip helps to reconfirm the FO at the instant of actual TSP real time.

Foramen ovalis identification through a bipolar potential reduction of the mapping catheter was applicable in only 69.6% of patients in our determination cohort, which is consistent with the finding of a previous study that observed low bipolar voltage in only half of FO mapping.^9^ Moreover, low-voltage zones may exist in persistent AF because of cardiac fibrosis unrelated to the FO.^9–11^ Moreover, the absence of FO mechanical protrusion in ∼25% of patients in our cohort, along with the finding of a previous study,^11^ is frequently due to a thick or fibrotic FO or previous TSP.^12^ Our study reveals that changes in the COI on the UEGM tip exhibited an additional value in determining the FO with a high success rate both in the derivation and the validation cohorts, even in such undetermined cases. Even a slight movement without the jump sign of the catheter tip in the 3D-EAMS can significantly alter the UEGM rather than the bipolar electrogram (see Supplementary material online, *Figure S4*). This may partially explain why our study found lower failure rates and complications than traditional fluoroscopy-guided TSP.^13^ This novel method of simply switching on the UEGM channel without any additional fee is both cost-effective and convenient.

### Patients who met at least one criterion had a high success rate in the pure three-dimensional electroanatomic mapping system–guided transseptal puncture procedure

The presence of the COI, which is widely used for pacemaker lead placement to address electrode pressure trauma against the endocardium,^17^ indicated firm contact between the needle tip and the muscular limbus. Similarly, COI disappearance or significant reduction was observed in the central FO due to the limited amount of myocardial tissue. These results are consistent with those of a previous TSP study performed under routine X-ray guidance,^18^ explained by the histological structure of the atrial septum.^19^

Intracardiac echocardiography is undoubtedly important for the safety and success of 3D-TSP. However, its necessity still warrants careful evaluation because of its high financial cost and resource availability, particularly in most routine cases where a combined UEGM tip with 3D-EAMS guidance can ensure safe TSP procedures.^7–11^ No previous studies have addressed the issue of balancing the security and cost of purely fluoroless 3D-TSP. Therefore, we propose a workflow with four criteria to individually determine eligible candidates while identifying which patients need ICE assistance. Our results indicate that in 78.2% of patients in the NFC group who do not meet any of the four criteria, it is challenging to perform TSP, and these patients require ICE assistance, compared with 2.6% of the OFC group. Conversely, patients meeting all four criteria achieved a 100% success rate, while those meeting at least one criterion in larger candidate groups attained a 97.4% success rate for pure 3D-TSP without additional imaging methods, including one difficult case of a patient with severe kyphoscoliosis and a patient with a minimum LA diameter of 18.8 mm. Above all, the eligible candidates for pure 3D-TSP were patients who met all four criteria. However, patients meeting at least one criterion were found suitable for attempting pure 3D-TSP with a high success rate, considering the issue of balance of security and cost, while those not meeting any of the four criteria required ICE guidance.

### Workflow and safety

Ancillary tools played pivotal roles, although the COI changes occurring on the UEGM tip were crucial for achieving a successful 3D-TSP. Preoperative CT and echocardiography were used to evaluate the cardiac structure, excluding a small minority of patients with disqualifying abnormalities of the heart. A rehearsal using an ablation catheter and subsequently with a needle was conducted to intra-operatively reassess the feasibility and safety of 3D-TSP by following the workflow. Intracardiac echocardiography or fluoroscopy may be considered to ensure safety in patients in whom anatomical abnormalities or difficulty in determining FO occur either with the catheter or with the UEGM tip.

### Limitation

The present study is limited in terms of replicability due to its single-centre design, reliance on experienced operators, and use of the CARTO 3 system for all procedures. Additionally, evaluation in a larger multicentre cohort with multiple operators is required before it is rolled out more widely. The results of the study apply to patients undergoing pre-procedural echocardiography and conventional CT (or low-dose CT in <18 years old). Moreover, thermal and non-thermal ‘single-shot’-based ablation techniques are increasingly used these days; thus, validating our method with any other mapping system would be important. However, the transseptal needle can easily be connected to any electrophysiology recording system; thus, no concern arises in this respect.

Despite these limitations, this study reveals the good safety and feasibility of a 3D-EAMS-guided TSP in combination with a UEGM tip. The workflow that is mainly based on this novel technique may provide benefits in terms of reducing procedural costs and eliminating the need for fluoroscopy. Additionally, it can be used in traditional TSP under routine fluoroscopic guidance, although it was originally designed for non-fluoroscopy.

## Conclusions

The COI changes on the UEGM tip when it was withdrawn from the muscular limbus into the FO were found to be a more reliable indicator for identifying the foramen ovalis compared with previously reported methodologies. The workflow mainly based on this novel technique could result in a safe and successful 3D-TSP without fluoroscopy. In this study, patients who met all four criteria mentioned previously were found suitable for 3D-TSP, while those who met none required ICE guidance.

## Supplementary Material

euae098_Supplementary_Data

## Data Availability

The data are available upon request from the authors.
