# Supplementary material for: Optimizing transseptal puncture guided by three-dimensional mapping: the role of a unipolar electrogram in a needle tip
Source: Europace. 2024 Apr 15;26(4):euae098. doi: 10.1093/europace/euae098 (PMC11050654; doi:10.1093/europace/euae098)
Supplement: euae098_Supplementary_Data [file euae098_supplementary_data.zip › supplementary file.docx]

**Supplemental Appendix**

Supplemental Figures

**Supplemental Figure 1**.Computerized tomographic scan can provide anatomic information for transseptal puncture.

**Supplemental Figure2.** A patient who failed to identify the FO by UEGM-tip.

**Supplemental Figure 3.** A patient with severe kyphoscoliosis successfully underwent 3D-TSP.

**Supplemental Figure 4.** COI exhibited as more reliable method to identify FO.

**Video 1.** A patient in sinus rhythm demonstrating successful fluoroless transseptal puncture utilizing UEGM-tip.

**Video 2.** A patient in atrial fibrillation demonstrating successful fluoroless transseptal puncture utilizing UEGM-tip.

**Supplemental Figure 1.**  **Computerized tomographic scan can provide anatomic information for**  **transseptal puncture.**


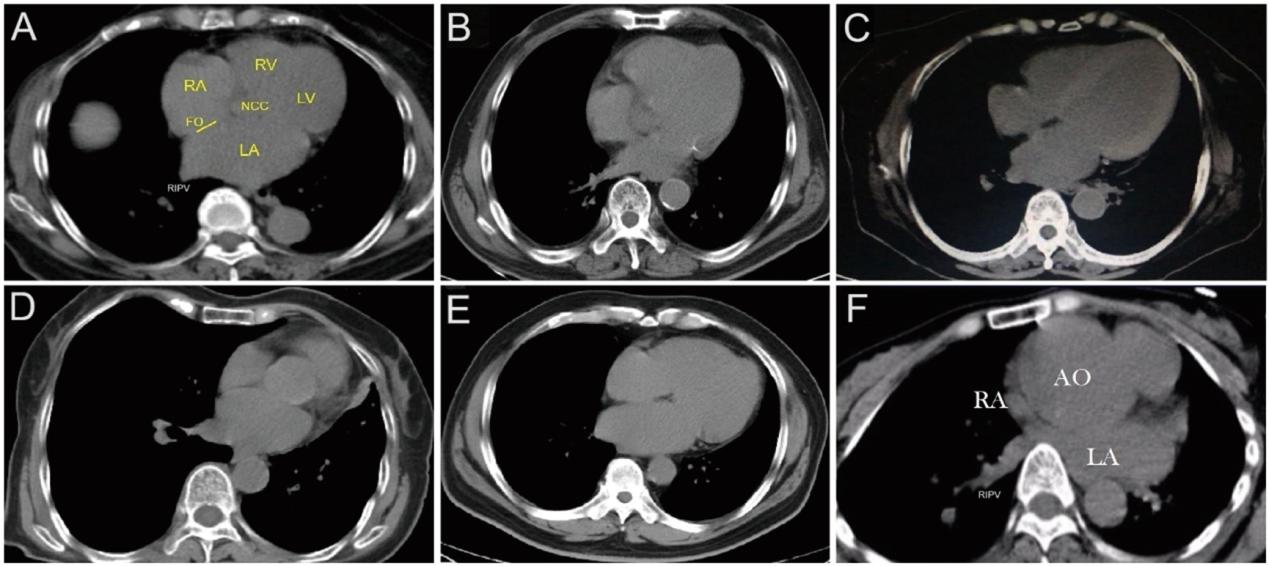


The feasibility of transseptal puncture was evaluated in a representative case using computerized tomographic scan to assess anatomic details, including the dimensions of the right and left atrium, orientation of the atrial septum, cardiac rotation, and adjacent aortic root size (A). Despite anatomical abnormalities such as lipomatous hypertrophy of the interatrial septum (B), interatrial septum with a narrow kissing area (C), and rotational abnormalities of the heart (D and E), all patients underwent successful transseptal puncture guided by electroanatomic mapping and needle tip unipolar electrogram without fluoroscopy except for one 83-year-old female (F). She had previously undiagnosed significant dilation of the ascending aorta resulting in cancellation of the planned transseptal and ablation procedure.

Abbreviations: AO = the root of the aorta; FO = foramen ovalis; LA = left atrium; LV = left ventricle; NCC = noncoronary cusp; RA = right atrium; RIPV = right inferior pulmonary vein; RV = right ventricle.

**S****upplemental Figure 2. A patient who failed to identify the FO by UEGM-tip.**


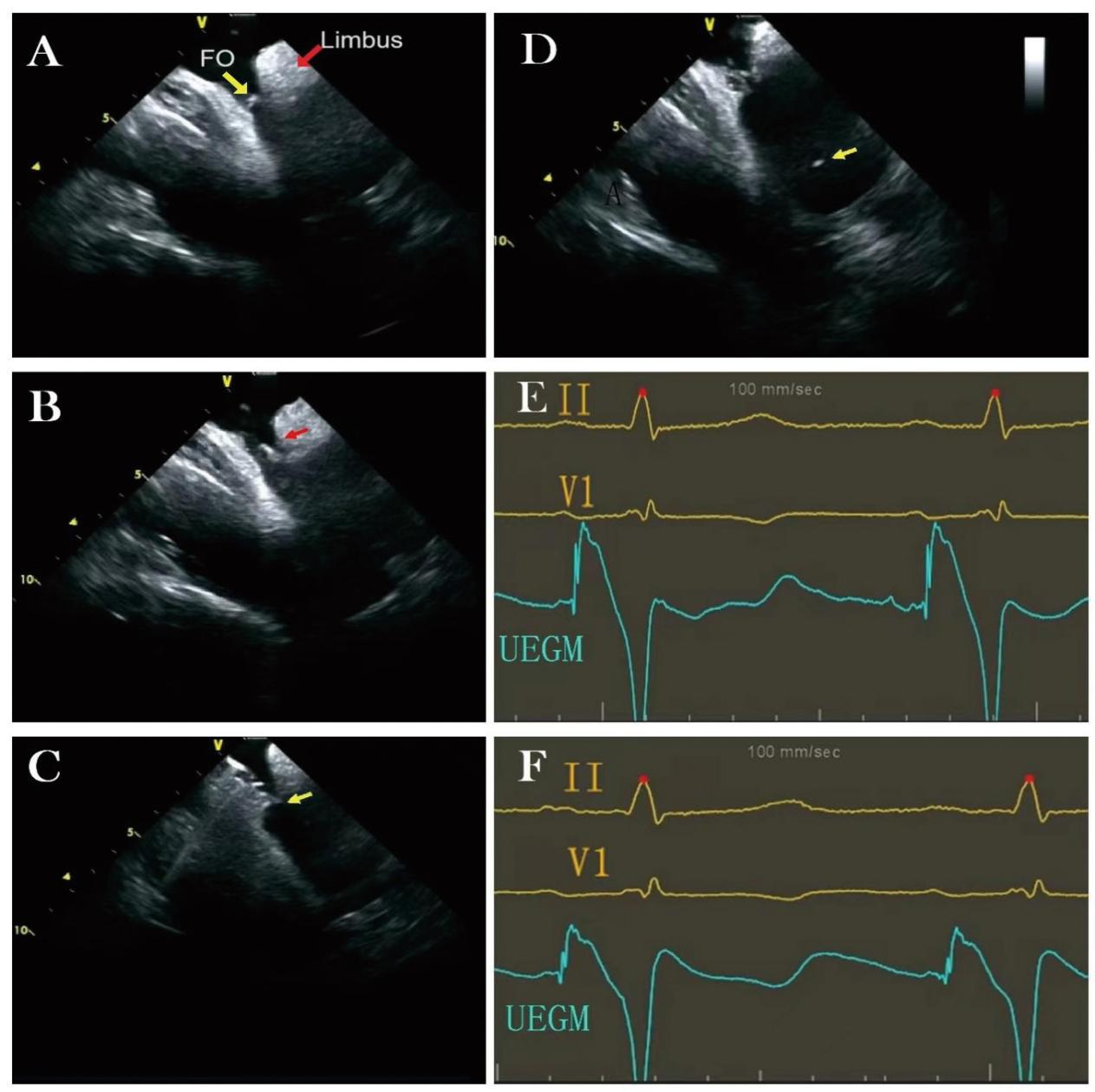
The intracardiac echo (A) displayed a patient with a thick limbus and a very small FO. During the TSP procedure, when the needle tip was from the muscular limbus of the FO (B, indicated by red arrow) to the central FO (C, indicated by yellow arrow), no obvious changes were observed in either spiked unipolar potentials or current of injury on UEGM-tip between the limbus (E) and FO (F). Successful TSP was achieved under intracardiac echo monitoring (D, needle tip indicated by yellow arrow). In this case, although the needle tip reached the FO, it also made contact with the muscular limbus, thereby neither spiked unipolar potentials nor current of injury changes could identify this particular FO.

Abbreviations: FO = foramen ovalis; TSP = transseptal puncture; (UEGM)-tip = (unipolar electrogram) derived from the transseptal needle tip.

**Supplemental Figure 3. A patient with severe kyphoscoliosis successfully underwent 3D-TSP.**


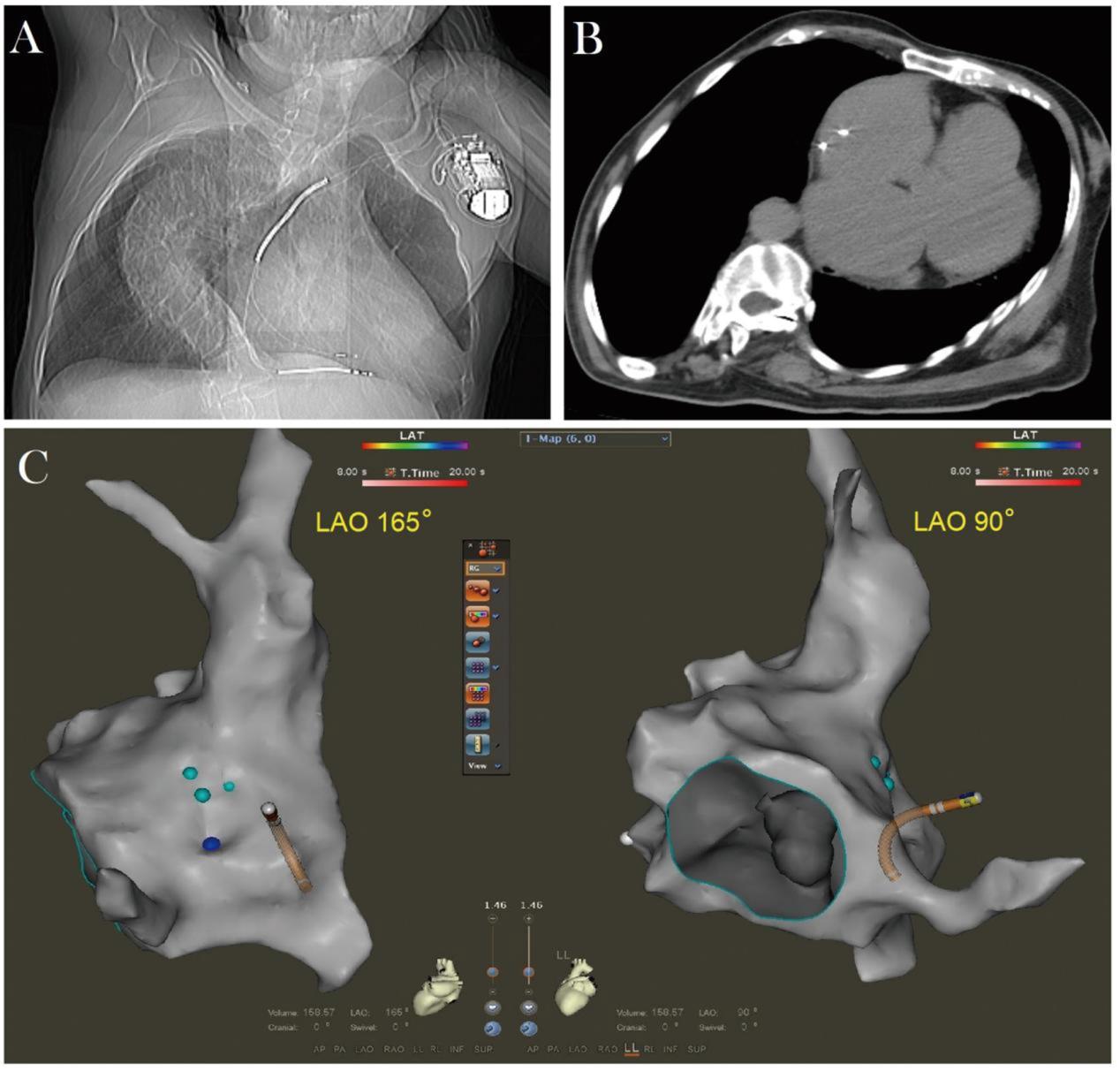
The Chest X-ray (A) and computed tomography scan (B) revealed severe kyphoscoliosis in a patient who went on to have successful transseptal puncture guided by electroanatomic mapping and unipolar electrogram on needle tip without the need for fluoroscopic or echocardiographic guidance (C).

Abbreviations: 3D-TSP = three dimensional mapping system guided transseptal puncture;

**Supplemental Figure 4.** **COI** **exhibited as a more reliable method to identify FO.**
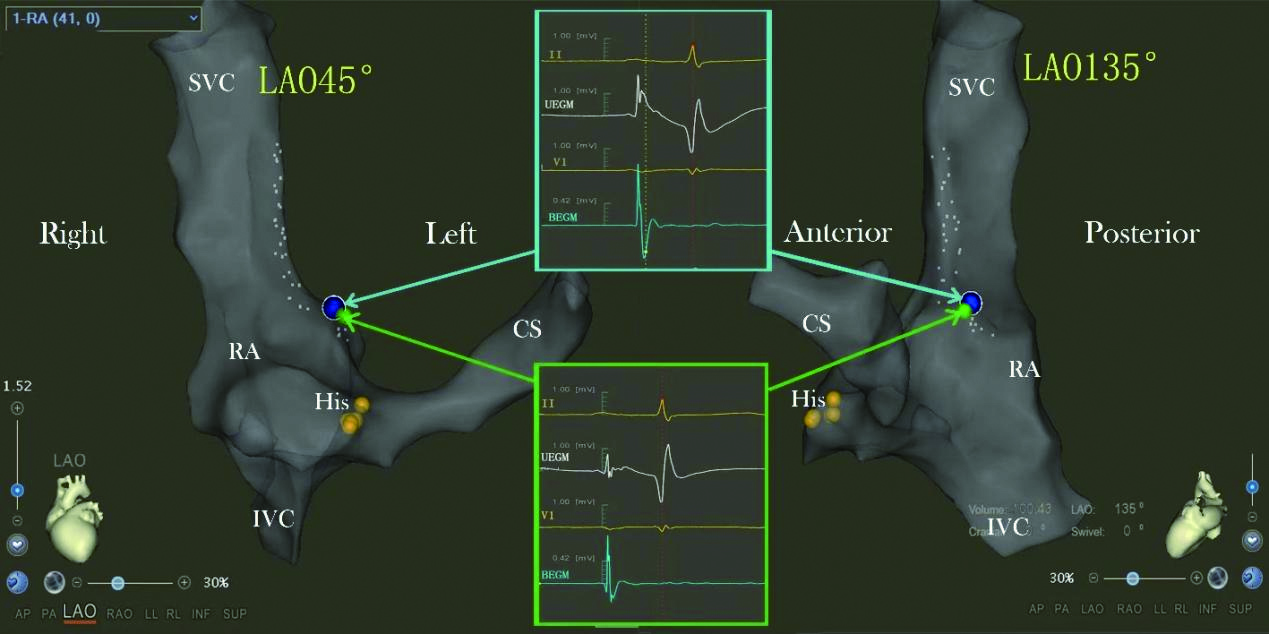
A slight movement of the ablation catheter tip from the muscular limbus (blue dot) to the central foramen ovalis (green dot) in the electroanatomic map could lead to significant changes in the morphology of the unipolar electrogram including change of the current of injury without obvious alteration of the amplitude of the unipolar or bipolar electrogram. Note that both dots were located in the protrusion area. The unipolar electrogram was recorded from the distal electrode of the ablation catheter, with an amplitude of 1.8mv at limbus (blue dot), while measuring 1.1mv at the fossa ovalis (green dot). For the bipolar electrogram, the amplitude was 1.79mv at the limbus (blue dot) was compared to 1.25mv at the foramen ovalis (green dot). Abbreviations: COI = current of injury. CS = coronary sinus; BEGM = bipolar electrogram; FO = fossa ovalis; His = His bundle; IVC = inferior vena cava; RA = right atrium; SVC = superior vena cava; UEGM = unipolar electrogram.

**Video 1. A patient in sinus rhythm demonstrating successful fluoroless transseptal puncture utilizing UEGM-tip.**

3D-electroanatomic mapping system guided transseptal puncture was successfully performed under the classical changes in UEGM-tip in a representative patient in sinus rhythm.

**Video 2.** **A patient in atrial fibrillation demonstrating successful fluoroless transseptal puncture utilizing UEGM-tip.**

3D-electroanatomic mapping system guided transseptal puncture was successfully performed under the classical changes in UEGM-tip in a representative patient in atrial fibrillation.
